# Identification of a Novel Metabolic Target for Bioactive Triterpenoids Biosynthesis in *Ganoderma lucidum*

**DOI:** 10.3389/fmicb.2022.878110

**Published:** 2022-05-09

**Authors:** Juan Xu, Yiyi Wang, Yi Zhang, Kehui Xiong, Xiaoyun Yan, Shiyu Ruan, Xueqian Wu

**Affiliations:** ^1^State Key Laboratory of Subtropical Silviculture, Zhejiang A&F University, Lin’an, China; ^2^Zhejiang Provincial Key Laboratory of Characteristic Traditional Chinese Medicine Resources Protection and Innovative Utilization, Zhejiang A&F University, Lin’an, China; ^3^National Ganoderma lucidum and Tetrastlgma hemsleyanum Industrial Innovation Alliance, Zhejiang A&F University, Lin’an, China; ^4^Zhejiang Wuyangtang Pharmaceutical Co., Ltd., Lishui, China

**Keywords:** *GlbHLH5* transcription factor, positive transcriptional regulator, *Ganoderma* triterpenoids, overexpression, RNA interference

## Abstract

Triterpenoids are crucial active ingredients of *Ganoderma lucidum* (*G. lucidum*) with various health benefits. Yet the low biosynthesis greatly restricts their industrial applications, novel metabolic engineering strategies are needed to further enhance *Ganoderma* triterpenoids production. Transcription factors play vital roles in the metabolic regulation of terpenoids, which are still scarce to study in *G. lucidum*. Herein, a transcription factor *GlbHLH5* (GenBank No. MZ436906.1) potential for metabolic regulation of *Ganoderma* triterpenes was identified for the first time. MeJA could increase *Ganoderma* triterpenoids biosynthesis, and *GlbHLH5* significantly responded to MeJA induction, suggesting *GlbHLH5* is a new target for *Ganoderma* triterpenoids overproduction. The regulatory effect of the newly identified target was further validated by homologous gene overexpression and silence in *G. lucidum*. It’s demonstrated that overexpression of *GlbHLH5* significantly increased triterpenoids accumulation and the key enzyme genes transcription in the biosynthetic pathway, while silencing it displayed the opposite effect, indicating *GlbHLH5* could positively regulate the triterpenoids biosynthesis by activating the synergistic expression of key enzyme genes in the biosynthetic pathway. Consequently, *GlbHLH5* was identified as a positive regulator and novel metabolic target for *Ganoderma* triterpenoids biosynthesis, it sheds new lights on the regulatory effect regulation and synthetic biology of *Ganoderma* triterpenoids.

## Introduction

*Ganoderma lucidum* (*G. lucidum*). Karst is a precious and famous large fungus as a food and medicine homologous. It is rich in *active* polysaccharides (Hu et al., 2019), triterpenoids (He et al., 2019), nucleosides (Chen Y. et al., 2012), sterols (Xu et al., 2021), and other substances with extremely high nutritional and medicinal values (Li et al., 2019a; Zhao et al., 2019). *Ganoderma* triterpenoids are the main active ingredients of *G. lucidum*. They are also known as ganoderic acids, which refer to a class of triterpenoids containing carboxyl and polyhydroxyl structures (Hu et al., 2020). As the crucial bioactive components, they have been recognized by the public for their functions of regulating the homeostasis of the body (Grienke et al., 2011), improving endocrine disorders (Zhao et al., 2012), and improving sleep quality (Yao et al., 2021). However, as a kind of secondary metabolites, the content of *Ganoderma* triterpenoids is relatively low in the raw material, which greatly restricts its development and application. It’s momentous to enhance active triterpenoids production by metabolically regulating their biosynthesis. Although improvement of *Ganoderm*a triterpenoids production had been achieved by optimizing fermentation substrates and process parameters, adding exogenous elicitors, and overexpressing the key enzyme genes in the triterpenoid’s biosynthetic pathway, novel metabolic engineering strategies are needed to further enhance *Ganoderma* triterpenoids production. Transcription factors play vital roles in the metabolic regulation of terpenoids, which are still scarce to study in *G. Lucidum* (Dhandapani et al., 2020; Wang et al., 2021). How to increase the biosynthesis of *Ganoderma* triterpenoids is a problem worthy of attention (Niego et al., 2021).

Jasmonic acids (JAs) are important signal molecules within plant cells, which regulate the expression of defense proteins and the synthesis of secondary substances through interactions with transcription factors (Li et al., 2021). The bHLH transcription factor is regarded as a core member of the JA signaling pathway. The genes involved in a variety of terpenoids biosynthetic pathways such as *SmTAT1* (Peng et al., 2020), *SmCYP98A14* (Di et al., 2013), *AaCYP71AV1* (Li et al., 2019b), and *AaDBR2* (Fu et al., 2021) are positively regulated by bHLH transcription factors. Therefore, bHLH is a positive regulator of terpenoids biosynthesis mediated by the JAs signal. It was found that exogenous MeJA could increase the biosynthesis of terpenoids in *G. Lucidum* by the signaling crosstalk between nitric oxide(NO) and reactive oxygen species (ROS) (Ren et al., 2010; Shi et al., 2020), but the mechanism of its transcriptional regulation remains unclear. Herein, we firstly discovered an endogenous bHLH member *GlbHLH5* responded to JAs in *G. lucidum*. Through bioinformatics analysis, and an excessive and silent expression of this gene in *G. lucidum* itself, we verified its role in the regulation of *Ganoderma* triterpenoids biosynthesis, and finally clarified the regulation mechanism of *Ganoderma* triterpenoid metabolism. It will provide new insights into the metabolic regulatory network associated with triterpenoids biosynthesis in *G. lucidum*, and define a new tool for improving *Ganoderma* triterpenoids production by burgeoning synthetic biology as well.

## Materials and Methods

### Strains and Culture Conditions

The *Ganoderma lucidum* strain (*G. lucidum* CGMCC5.26) used in this experiment was purchased from the Institute of Microbiology, Chinese Academy of Sciences, the DH5a competent cells were purchased from Vazyme, and AH109 competent cells were purchased from Shanghai Weidi Biotechnology Co., Ltd. The pGBKT7 vector was presented by Dr. Bingcong Xing from Zhejiang A&F University.

Fungal complete medium (CYM) and fungal protoplast cell wall regeneration medium (MYG) were used to cultivate *G. lucidum*. CYM medium was composed of 1% maltose, 2% glucose, 0.2% yeast extract, 0.2% peptone, 0.05% MgSO_4_⋅7H_2_O, 0.05% K_2_HPO_4_ (Zhou et al., 2014). MYG medium was composed of 1% glucose, 0.5% maltose, 0.5% yeast powder, 2% agar powder, dissolved in mannitol. LB medium: 1% tryptone, 0.5% yeast powder, 1% NaCl, 2% agar powder (solid), pH = 7.0.

### Cloning of *GlbHLH5* Gene Sequence

The primers were designed according to the CDS region of the *GlbHLH5* gene (Supplementary Table 1) and synthesized by Sangon Biotech. The total RNA of *Ganoderma lucidum* hyphae was extracted according to the instructions of the column-type fungal total RNA extraction and purification kit (Sangon Biotech, Shanghai, China). Agarose gel electrophoresis was used to detect the integrity of the total RNA, and a micro-ultraviolet spectrophotometer was used to detect the concentration and purity. Following the instructions of the PrimeScript™ RT Master Mix kit (Sangon Biotech, Shanghai, China), the total RNA was reversed into cDNA which was further used as a template for PCR amplification of the coding region of the *GlbHLH5* gene, and the PCR amplification system was as follows: PrimeSTAR Max Premix (2X) 25 μL, *GlbHLH5*-F 2.5 μL, *GlbHLH5*-R 2.5 μL, cDNA template 1.0 μL, ddH_2_O 19 μL. PCR was performed according to the manufacture’s instruction of PrimeSTAR^®^ Max DNA Polymerase (Takara, Japan) using the following protocol: 98^°^C, 5 min, 1 cycle; 98^°^C, 10 s, 55^°^C, 30 s, 72^°^C, 1 min, 30 cycles; 72^°^C, 5 min. After the PCR products were detected by 1.5% agarose gel electrophoresis, they were sent to Zhejiang Youkang Biotechnology Co., Ltd. for sequencing.

### Bioinformatics Analysis of *GlbHLH5*

A bHLH gene *GlbHLH5* (GenBank No. MZ436906.1) was obtained by comparing the *G. lucidum* genome database^1^ and the Pfam database (number: PF00010) (Chen S. et al., 2012; Qian et al., 2013; Finn et al., 2016; Chen X. et al., 2021). Online analysis software ORF Finder and Conserved Domain-search of the NCBI database were used to analyze the open reading frame (ORF) and conserved domains of the gene. ProtParam was performed to analyze the physicochemical properties of the *GlbHLH5* encoded protein. At last, highly similar protein sequences of other species were downloaded from the NCBI protein database, and a phylogenetic tree was constructed to perform the phylogenetic analysis by MEGA 7.0. PlantCARE was used to perform online promoter cis-acting element analysis on the nucleic acid sequences of 2 000 bp upstream of three key genes *HMGR* (GenBank No. EU263989) (Shang et al., 2008), *SQS* (GenBank No. DQ494674) (Zhao et al., 2007), and *LS* (GenBank No. GQ169528) (Shang et al., 2010) in the triterpenoid synthesis pathway of *Ganoderma lucidum*, and TBtools software was used for visual display.

### Prokaryotic Expression of *GlbHLH5*

The sequenced gene was translated, and the properties of the protein encoded by the *GlbHLH5* gene were analyzed. Using the method of homologous recombination, the *GlbHLH5* gene was integrated into the pET-32a vector, and the recombinant plasmid was transferred into *Escherichia coli DH5a* competent cells, cultured at 37°C overnight, and a single colony was picked for PCR verification and sent to the company for sequencing. Plasmid extraction was performed on the correctly sequenced recombinant-positive strains, and the plasmid DNA was transferred into *E. coli BL21 (DE3)* competent cells, cultured overnight at 37°C, and single colonies were picked and inoculated in Amp-resistant LB liquid medium, and then cultured at 37°C and 200 r⋅min^–1^ until the bacterial density (OD600) reached 0.8. Subsequently, 0.5 mmol⋅L^–1^. IPTG was added to induce 12 h at 16, 25, and 37°C, and the bacterial liquid was collected and detected by SDS-PAGE.

### Transcriptional Activation of *GlbHLH5*

To investigate the transcriptional activation of *GlbHLH5*, pGBKT7 (pBD) was used to construct yeast one-hybrid system pBD-*GlbHLH5* vector by *Eco*RI and *Bam*HI. After the sequencing results were correct, the plasmid pBD-*GlbHLH5* and the positive control pGBKT7 were transformed into yeast AH109, respectively. The constructed plasmid was coated on the defect medium SD/-Trp, cultured at 30°C for 3 days, and then transferred to SD/-Trp/-His/-Ade + x-a-Gal for color reaction.

### Analysis of MeJA Induced Expression Pattern of *GlbHLH5*

The 50 μmol L^–1^ MeJA was added to the fermentation broth of *G. lucidum* cultured for 5 days, and no MeJA treatment was used as a control. The triterpenoid content of *G. lucidum* was determined at 0, 2, 4, 6, 12, 24, and 48 h after MeJA induction. The relative expression of *GlbHLH5* was determined by qRT-PCR, and the response of the key enzyme genes in the triterpenoid’s biosynthetic pathways to MeJA induction was also investigated. Each experiment was repeated in three replicates, and the data are all expressed as mean ± SD (Gharari et al., 2020).

### Subcellular Localization of *GlbHLH5* Protein

The *GlbHLH5* sequence and overexpression vector pBARGPEI were analyzed, and primers containing *Bam*HI and *Eco*RI sites were designed (Supplementary Table 1). The pBARGPEI was digested with *Bam*HI and *Eco*RI, and recovered and purified for homologous recombination with *GlbHLH5* containing homologous arms (Song et al., 2021). The recombinant product was transferred into *E. coli DH5a* competent cells and cultured overnight at 37°C. Single colonies were picked for PCR verification of bacterial solution and sent to the company for sequencing. The plasmid was extracted from the bacterial solution with correct sequencing. The strains WT, pBARGPEI-GFP and pBARGPEI-*GlbHLH5*-GFP were inoculated in the fungal medium containing CaCl_2_, and the sterilized coverslip was inserted into the medium at 1 cm around the inoculation block. After the mycelia grew on the coverslip, the coverslip was stained with DAPI, and rinsed with PBS for several times. The positions of GFP protein, *GlbHLH5*-GFP fusion protein and nucleus in cells were observed by inverted fluorescence microscope.

### Construction of *GlbHLH5* Overexpression Vector

According to the *GlbHLH5* sequence and the overexpression vector pBARGPEI, primers containing restriction sites *Bam*HI and *Eco*RI (Supplementary Table 1) were designed. PBARGPEI was digested with *Bam*HI and *Eco*RI with double restriction enzymes, recovered and purified, and then homologously recombined with *GlbHLH5* containing homology arms. The recombined product was transferred to *E. coli DH5a* competent cells, cultured overnight at 37°C, and the single colonies were picked on Amp resistant plates. Then, bacterial liquid PCR and sequencing were performed to verify whether the recombinant vector was successfully constructed. At last the recombinant plasmids were transformed into protoplast of *G. lucidum*.

### Construction of *GlbHLH5* Gene Silencing Vector

According to pSilent-1 plasmid and *GlbHLH5* silencing sequences, suitable restriction sites were selected to design the primers (Supplementary Table 1). The pSilent-1 and *GlbHLH5* silencing sequences were double digested with *Xhol* and *Hin*dIII, and the digested products were ligated and transformed and sequenced to form the intermediate vector pS-1. Then, the intermediate vector and *GlbHLH5* reverse silencing sequence were doubly digested with *Sph*I and *Kpn*I, and the *GlbHLH5* reverse silencing sequence was insert into the downstream of the IT sequence of the vector to finally construct the pSilent-*GlbHLH5* recombinant plasmid with an inverted repeat “hairpin structure.”

### Protoplast Transformation and Transformant Verification

The overexpression and silencing vectors constructed above were transferred into *G. lucidum* protoplasts by a PEG-mediated method (Liu and Friesen, 2012), and the transformants were screened by hygromycin (G-Clone, Beijing, China) resistance, and the well-growing strains were further identified by colony PCR and the detection of the *GlbHLH5* gene expression.

### qRT-PCR and Triterpenoid Content Detection

The expression levels of *GlbHLH5*, *HMGR*, *SQS*, and *LS* genes were determined by qRT-PCR with *18S* rRNA as the internal reference gene, and the primers used were shown in Supplementary Table 1. qRT-PCR was performed according to the manufacture’s instruction of ChamQ Universal SYBR qPCR Master Mix (Vazyme, Nanjing, China) using the following protocol: 95°C, 30 s, 1 cycle; 95°C, 10 s, 60°C, 30 s, 40 cycles. Salkowski reaction was firstly used for qualitative detection of triterpenoid content in *G. lucidum*, and further quantitative determination was conducted by the vanillin-perchloric acid chromogenic method (Chen Z. et al., 2021).

### Statistical Analysis

GraphPad Prism 8.0 software was used for statistical analysis, in which the qRT-PCR results used *18S* rRNA as an internal reference, and the relative expression level of genes was calculated by the 2^–△△^ *^Ct^* method, and then the t-test was used to analyze the significance of the difference. “*” means the difference is significant (*P* < 0.05) and “^**^” means the difference is extremely significant (*P* < 0.01).

## Results

### Cloning and Bioinformatics Analysis of *GlbHLH5*

**FIGURE 1:**
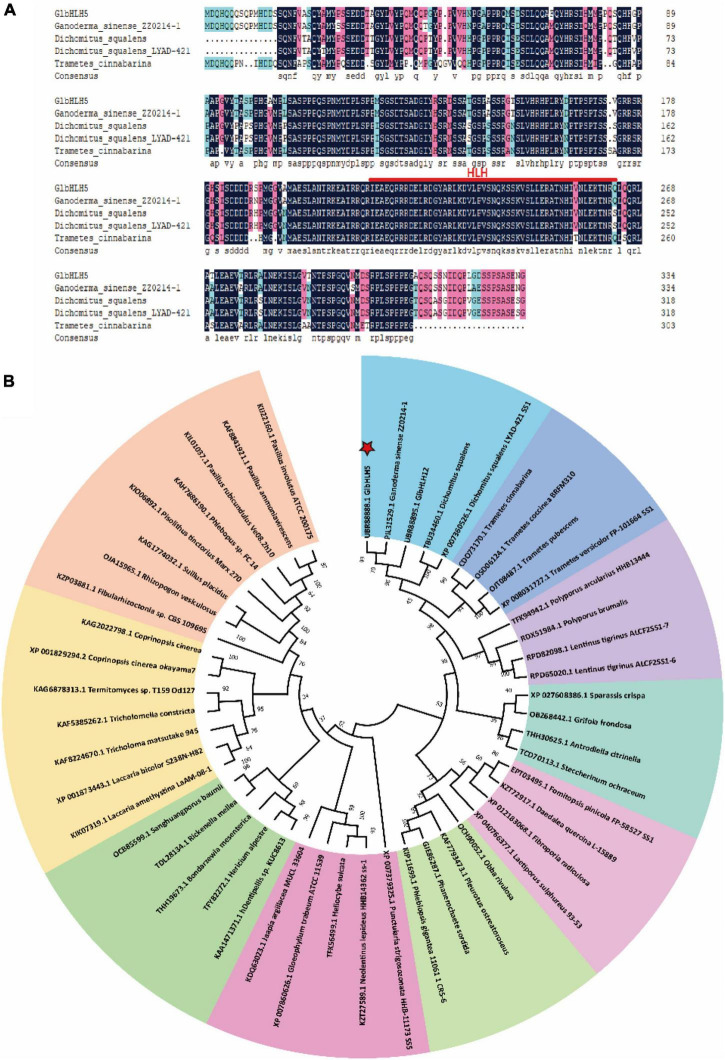
Bioinformatics analysis of GlbHLH5. **(A)** The comparison analysis of the amino acid sequence of *GlbHLH5* and other homologous proteins. **(B)** The adjacent tree of protein homologs, the percentage value of the leader program for 1,000 repetitions is displayed at the branch point.

The cDNA of *G. lucidum* was used as a template for PCR amplification. After sequencing, the full length of the *Ganoderma* bHLH transcription factor cDNA was 1008 bp, and the sequence had been uploaded to the NCBI database. The obtained gene sequence number was MZ436906.1 and named *GlbHLH5*. The relative molecular weight of *GlbHLH5* is 85113.08, the theoretical PI value is 4.98, and it could encode a protein with a molecular weight of about 36.58 kDa. The SMART analysis and multiple comparisons of amino acids showed that it contained an HLH domain composed of 51 amino acids. The phylogenetic analysis indicated that *GlbHLH5* was closely related to *Ganoderma sinensis* transcription factor *GsPIL31529.1* (Figure 1).

### Structure Prediction and Prokaryotic Expression of *GlbHLH5* Protein

**FIGURE 2:**
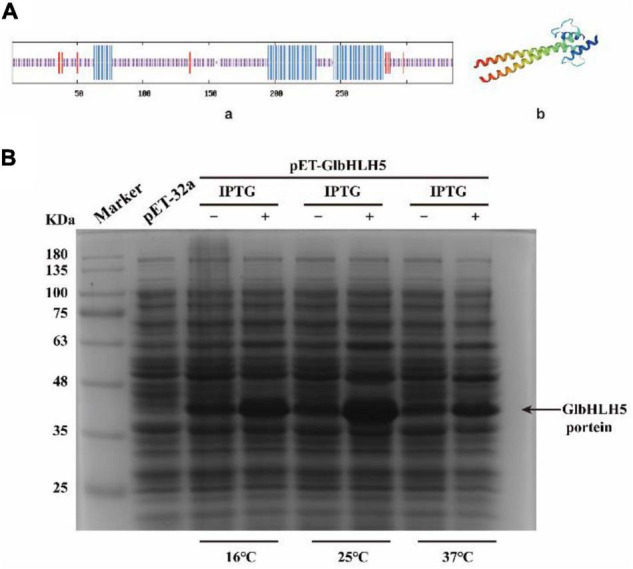
Structure prediction and prokaryotic expression of *GlbHLH5* protein. **(A)** Secondary (a) and three-dimensional (b) structure prediction of *GlbHLH5* Protein. **(B)** Prokaryotic expression of *GlbHLH5* Protein.

On-line protein analysis showed that there were three secondary structures of *GlbHLH5* gene. The proportion of α-helix (blue), extended strand (red) and random coil (purple) were 26.57, 5.97, and 67.46%, respectively (Figure 2A-a). SWISS-MODEL was used to predict the three-dimensional structure of *GlbHLH5* protein online. The three-dimensional structure was mainly α-helix and random coil, which was consistent with the predicted secondary structure of *GlbHLH5* protein (Figure 2A-b). Subsequently, the complete coding sequence of *GlbHLH5* was cloned into the pET-32a vector and transformed into *DH5a* competent cells. The recombinant plasmid pET-32a-*GlbHLH5* was sequenced and extracted. Then, the recombinant plasmid pET-32a-*GlbHLH5* was transformed into *E. coli BL21 (DE3)* competent cells. The empty vector pET-32a was used as the control. The recombinant cells were collected after IPTG induction for 12 h. After centrifugation, the cells were suspended in Tris-HCl for ultrasonic fragmentation and lysis. The supernatant was taken for SDS-PAGE detection after denaturation. The results showed that the pET-32a-*GlbHLH5* strain inducted by IPTG for 12 h could all express the protein with a size of about 36.58 KDa (Figure 2B). With the increase of induction temperature, the protein expression levels firstly increased and then decreased, and the expression level of the target protein was the highest under the induction condition of 25°C.

### Transcriptional Activation Analysis of *GlbHLH5*

**FIGURE 3:**
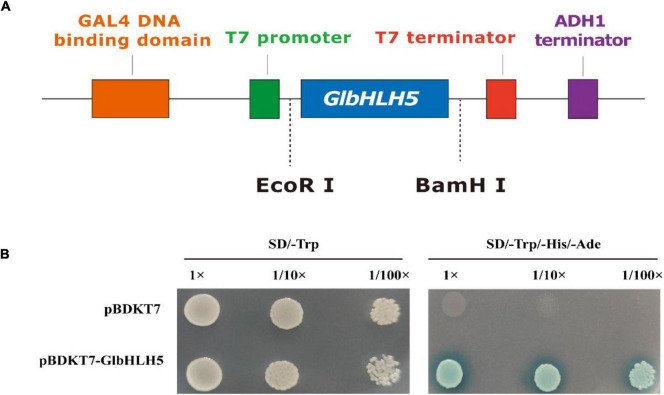
Transcriptional activation analysis of *GlbHLH5.* **(A)** The construction of the vector pGBKT7-*GlbHLH5*. **(B)** Transcription activation activity of *GlbHLH5*. 3-AT was added at a final concentration of 0.5 mmol L^–1^.

The complete coding sequence of *GlbHLH5* gene was cloned into the pGBKT7 (pBD) vector using the yeast single hybridization system, and the constructed pBD-*GlbHLH5* vector was identified by colonies PCR and sequencing. The sequenced recombinant plasmid was transformed into the yeast *AH109* strain to observe its growth on the Kan-resistant plate. The results showed that pBD empty vector and pBD-*GlbHLH5* transformants could grow well on SD/-Trp plates, while only pBD-*GlbHLH5* could grow well on SD/-Trp/-His/-Ade solid medium supplemented with 3-AT (aminotriazole) (Figure 3). Because AT is His synthase inhibitor, it can reduce the leakage expression of His synthase in yeast AH109 strain and improve the selection conditions. The above results show that *GlbHLH5* has strong transcriptional activation activity.

### Induced Expression Pattern of *GlbHLH5* by MeJA

**FIGURE 4:**
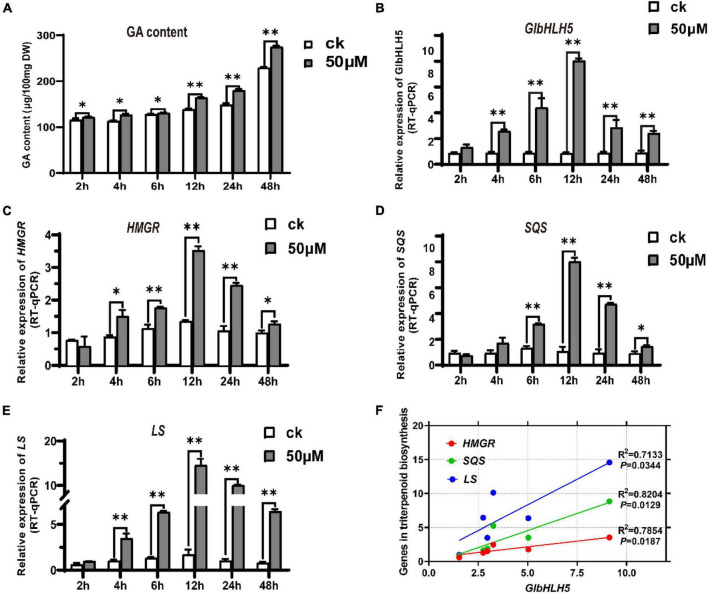
MeJA induced triterpenoids accumulate and expressions of *GlbHLH5* along with key genes in the triterpenoids biosynthetic pathway in *Ganoderma lucidum.* **(A)** Changes of triterpenoids content induced by MeJA in *G. lucidum*. **(B–E)** Relative expression levels of *GlbHLH5* and key genes in the triterpenoids biosynthetic pathway. **(F)** Correlation analysis between *GlbHLH5* and key genes in triterpenoids biosynthesis pathway. “*” means the difference is significant (*P* < 0.05) and “**” means the difference is extremely significant (*P* < 0.01).

Compared with the control group, adding exogenous MeJA significantly increased the content of *Ganoderma* triterpenoids, and the difference was extremely significant after 12 h of induction (*P* < 0.01) (Figure 4A). To further clarify whether *GlbHLH5* participates in the regulation of *Ganoderma* triterpenoids biosynthesis by responding to jasmonic acid signals, qRT-PCR was used to determine the response of *GlbHLH5* and key genes *HMGR*, *SQS*, *LS* in the biosynthetic pathway of *Ganoderma* triterpenoids to MeJA induction. After 4 h of induction with 50 μmol L^–1^ MeJA, the expression of *GlbHLH5* increased significantly (*P* < 0.01), which was twice that of the control group (Figure 4B); compared to the control group, the expression of *HMGR* increased significantly (*P* < 0.05) after 4 h of induction, and reached its peak at 12 h of induction (Figure 4C); after 6 h of induction, the expression of *SQS* was extremely significantly (*P* < 0.01) higher than the control group, the expression level reached its peak at 12 h after induction (Figure 4D); the expression of *LS* was extremely significantly (*P* < 0.01) higher than the control group after 4 h of induction (Figure 4E). To sum up, the relative expression levels of the three key genes in the triterpenoids biosynthetic pathway all reached their peak values at 12 h after MeJA induction, which was consistent with the induced expression pattern of *GlbHLH5*. At the same time, the correlation analysis showed that the expression of *GlbHLH5* has a very significant (*P* < 0.01) positive correlation with the three key genes *HMGR*, *SQS*, and *LS* (Figure 4F). Promoter cis-acting elements analysis of the three key genes shown that they all contained multiple G-box, which is the binding site of bHLH transcription factors. It was suggested that *GlbHLH5* transcription factor might be involved in triterpenoid biosynthesis in *G. lucidum* by regulating *HMGR*, *SQS*, and *LS* gene expressions. The regulatory effect of *GlbHLH5* in *Ganoderma* triterpenoids biosynthesis will be further analyzed and verified by transgenic technology.

### Subcellular Localization of *GlbHLH5*

**FIGURE 5:**
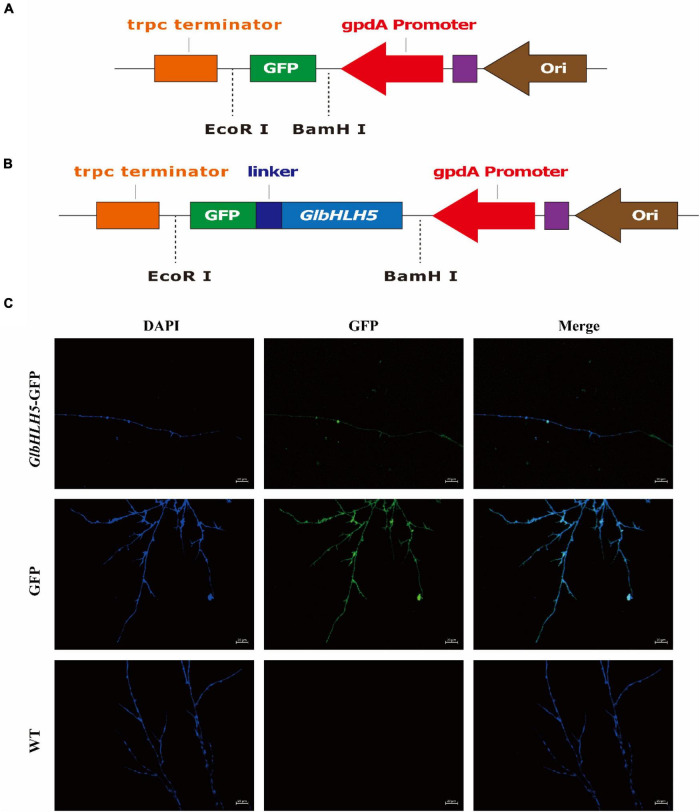
Subcellular localization analysis of *GlbHLH5.* **(A)** Construction of the vector pBARGPEI-GPF. **(B)** Construction of the vector pBARGPEI-*GlbHLH5*-GPF. **(C)** Subcellular localization of *GlbHLH5* protein in *Ganoderma lucidum*. DAPI, DAPI nuclear staining; GFP, the field of view under green excitation fluorescence; Merge: the overlapping field of view of DAPI and GFP.

The online software WoLF PSORT was used to analyze *GlbHLH5* transcription factor, and the prediction showed that it was mainly located in the nucleus. The fluorescent protein was observed by laser confocal microscope, and the pBARGPEI-GFP was used as the control group, and the pBARGPEI-*GlbHLH5*-GFP was used as the experimental group. The results showed that in the presence of Ca^2+^, the nucleus of pBARGPEI-*GlbHLH5*-GFP strain had obvious fluorescence, while that of WT strain had no fluorescence (Figure 5). It was indicated that *GlbHLH5* transcription factor mainly located in the nucleus of *G. lucidum*, which was consistent with the predicted results, indicating that *GlbHLH5* plays a role in the nucleus.

### Effect of *GlbHLH5* Overexpression on the *Ganoderma* Triterpenoids Biosynthesis and Key Genes Expression of Biosynthetic Pathway

**FIGURE 6:**
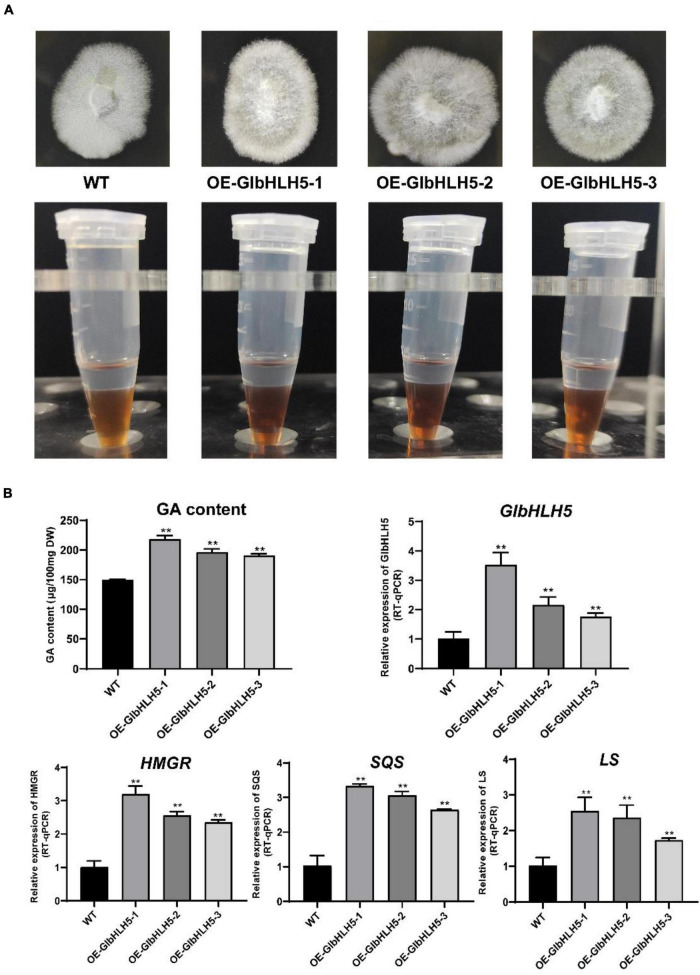
Phenotypic and relative gene expressions analysis of the *GlbHLH5* overexpression strains. **(A)** The phenotypes of the three *GlbHLH5* overexpression and the control lines of *Ganoderma lucidum* and the coloration differences between extracts of different lines. **(B)** The quantitative analysis of triterpenoids and the key genes transcriptions of the biosynthetic pathway in the *GlbHLH5* overexpression and the control lines. “**” means the difference is extremely significant (*P* < 0.01).

Transgenic *Ganoderma lucidum* strains overexpressing *GlbHLH5* were generated by a PEG-mediated genetic transformation system. The overexpression lines were selected by hygromycin resistance. PCR amplification was also conducted to verify the positive transformants. A 580 bp gpd promoter of the vector was amplified in the *GlbHLH5*-overexpressed positive transformants but not in the wild strain. It was demonstrated in Figure 6A that *GlbHLH5* overexpressed *G. lucidum strains* could grow well on the hygromycin plate. As for the biosynthesis of *Ganoderma* triterpenoids, it was shown that the color developing of three overexpression lines (OE-*GlbHLH5*-1, OE-*GlbHLH5*-2, and OE-*GlbHLH5*-3) was deeper than that of the wild type in Salkowski qualitative reaction, visually manifesting the triterpenoid content of the three overexpression lines might be higher than that of the wild type. Further quantitative analysis by the vanillin-perchloric acid chromogenic method revealed that the triterpenoid content of OE-*GlbHLH5*-1, OE-*GlbHLH5*-2, and OE-*GlbHLH5*-3 lines increased by 45, 37, and 21%, respectively (Figure 6B) compared with the wild type. qRT-PCR confirmed that much higher expression levels of *GlbHLH5* were observed in the three overexpression lines. Moreover, the transcript levels of the key genes *HMGR*, *SQS*, and *LS* in the biosynthetic pathways of *Ganoderma* triterpenoids were all upregulated in *GlbHLH5* overexpression lines compared with those in the wild type. As shown in Figure 6B, the relative expressions of *HMGR*, *SQS*, and *LS* reached the highest in line OE-*GlbHLH5*-1, which were 3.16, 3.23, and 2.62 fold of the wild type, respectively. Last but not the least, the variations of the pathway genes transcription were consistent with the accumulation of *Ganoderma* triterpenoids, and the expression levels of *GlbHLH5* as well, indicating that overexpression of *GlbHLH5* transcription factor could increase the triterpenoid content by activating the biosynthetic pathways of triterpenoids in *G. lucidum*.

### Effect of Silencing *GlbHLH5* on the *Ganoderma* Triterpenoids Synthesis and the Key Genes Expressions of Biosynthetic Pathway

**FIGURE 7:**
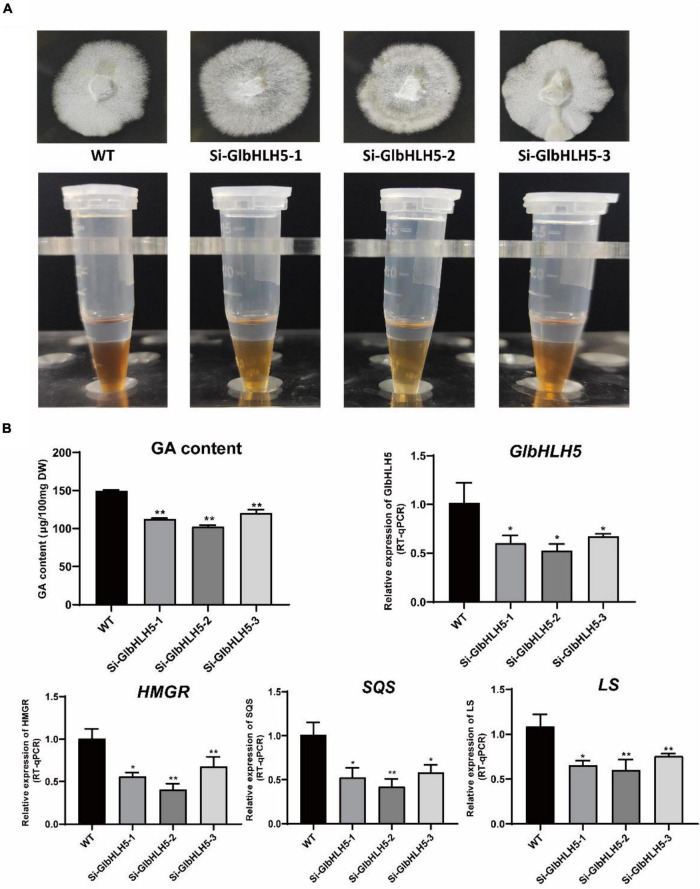
Phenotypic and gene expressions analysis of the *GlbHLH5* silencing strains. **(A)** The phenotypes of the three *GlbHLH5* silencing and the control lines of *Ganoderma lucidum* and the coloration differences between extracts of different lines. **(B)** The quantitative analysis of triterpenoids and the key genes transcriptions of the biosynthetic pathway in the *GlbHLH5* silencing and the control lines. “*” means the difference is significant (*P* < 0.05) and “**” means the difference is extremely significant (*P* < 0.01).

Three stably inherited *GlbHLH5* silenced strains were obtained through PEG-mediated multiple-resistance screening on Hyg plates, and the Hyg gene of the vector was amplified from the *GlbHLH5*-silenced positive transformants, yet not from the wild strain. As shown in Figure 6B, the relative expression levels of *GlbHLH5* gene in the three selected *GlbHLH5* silencing lines (Si-*GlbHLH5*-1, Si-*GlbHLH5*-2, and Si-*GlbHLH5*-3) were significantly lower than those in wild type (*P* < 0.05). The effect of silencing *GlbHLH5* on the *Ganoderma* triterpenoids synthesis and the key genes expressions of biosynthetic pathway was completely opposite to that of *GlbHLH5* overexpression. Compared with the wild type, the accumulation of *Ganoderma* triterpenoids decreased in *GlbHLH5*-silenced lines, as the Salkowski qualitative reaction showed that the color developing of the three silencing strains was shallower than that of the wild type (Figure 7A). Further quantitative analysis by the vanillin-perchloric acid chromogenic method showed that the triterpenoids content of Si-*GlbHLH5*-1, Si-*GlbHLH5*-2, and Si-*GlbHLH5*-3 decreased by 24, 31, and 19%, respectively. To further verify the function of *GlbHLH5* on regulating *Ganoderma* triterpenoids synthesis, the key genes expressions of their biosynthetic pathways were determined by quantitative RT-PCR. It was revealed that silencing of *GlbHLH5* gene leaded to down-regulation of the biosynthetic pathways of *Ganoderma* triterpenoids. The relative expression levels of *HMGR*, SQS, and *LS* genes in lines Si-*GlbHLH5*-1, Si-*GlbHLH5*-2 and Si-*GlbHLH5*-3 were all significantly lower than those in wild type (*P* < 0.01) (Figure 7B), and the variation in triterpenoids biosynthetic pathway gene transcription was generally consistent with *GlbHLH5*. The results showed that silencing *GlbHLH5* transcription factor could reduce the biosynthesis of triterpenoids in *G. lucidum*, which further proved that *GlbHLH5* transcription factor was a transcription factor involved in the regulation of *Ganoderma* triterpenoids biosynthesis.

## Discussion

The bHLH transcription factor family has many members and diverse functions. It is the second-largest transcription factor in plants (Carretero-Paulet et al., 2010). It plays an important regulatory role in plant growth and development (Verma et al., 2020), morphogenesis (Conway et al., 2010), stress resistance (Zhao et al., 2020), and secondary metabolism (Chezem and Clay, 2016). In the regulation of secondary metabolism, bHLH combines with the cis-acting elements in the promoter region of the target gene at the transcription level to activate the expression of the related genes. Its important role is to regulate the coordinated expression of multiple genes in the biosynthetic pathways of secondary metabolism, so as to globally activate secondary metabolic pathways and promote the biosynthesis of secondary metabolites (Zhou and Memelink, 2016). Studies have shown that bHLH is an important kind of transcription factor regulating terpenoid metabolism. *AaTAR2* (Zhou et al., 2020), *AaMYC2* (Chen et al., 2017), and *AaDELLA* (Shen et al., 2015) in *Artemisia annua* can promote the biosynthesis of artemisinin. The bHLH transcription factors *BIS1* (Pan et al., 2018) and *CrMYC2* (Zhang et al., 2011) in *Catharanthus roseus* regulate the synthesis of terpenoid alkaloids in response to the induction of MeJA. *SmbHLH51* (Wu et al., 2018), *R2R3-MYB* (Li and Lu, 2014), and *SmMYC2* (Yang et al., 2017) in *Salvia* can positively regulate the metabolism of tanshinone substances. Recently, an increasing number of bHLH proteins have been identified from diverse fungi, and they involve in a variety of biological processes, such as mycelial growth, cellular differentiation, sporulation, virulence, and secondary metabolism (Sailsbery et al., 2012), but bHLH family in *G. lucidum* are rarely studied. Whether *Ganoderma* bHLH can regulate the synthesis of *G. lucidum* triterpenoids is still unclear.

In this study, the *GlbHLH5* gene was cloned and proved to contain the bHLH conserved domains and DNA binding sites (Figure 1A), which was basically consistent with the sequences reported by Chen S. et al. (2012). Phylogenetic tree analysis found that *GlbHLH5* and many homologous proteins in Basidiomycota were clustered on the same phylogenetic branch except for *Boletus edulis* and *Pleurotus ostreatus* (Figure 1B), indicating that the *Ganoderma* bHLH transcription factors have species specificity.

MeJA can significantly induce the synthesis of *Ganoderma lucidum* triterpenoid but the mechanism of its transcriptional regulation remains unclear. Using qRT-PCR, we analyzed the expression patterns of *GlbHLH5* and the three key genes of *Ganoderma* triterpenoid biosynthetic pathway, *HMGR*, *SQS* and *LS*, under the induction of MeJA. As shown in Figure 4, the expressions of *GlbHLH5, HMGR*, *SQS*, and *LS* were all significantly induced by MeJA, and the consistent induced expression pattern between *GlbHLH5* and the three key genes of triterpenoids biosynthetic pathway indicated that *GlbHLH5* may regulate the transcription of the key genes *HMGR*, *SQS*, and *LS* in the triterpenoid metabolism pathway to promote the biosynthesis of *Ganoderma* triterpenoids.

**FIGURE 8:**
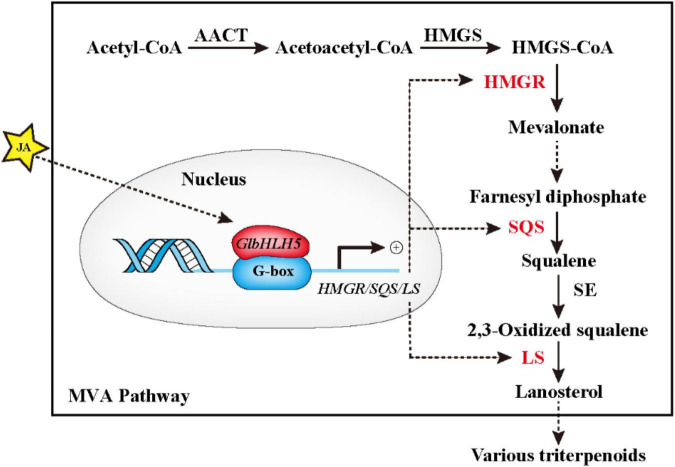
Schematic of the predicted regulatory effect of *GlbHLH5* in *Ganoderma* triterpenoids biosynthesis. The solid box represents the main pathway (MVA pathway) of Ganoderma triterpenoids. AACT, acetyl-CoA acetyltransferase; HMGS, 3-hydroxy-3-methylglutaryl-CoA synthase; HMGR, 3-hydroxy-3-methylglutaryl-CoA reductase, SQS, squalene synthase; SE, squalene monooxygenase; LS, lanosterol synthase.

We further used transgenic technology to verify the regulatory effect of *GlbHLH5*, and found that the overexpression of *GlbHLH5* in *G. lucidum* significantly increased the accumulation of *Ganoderma* triterpenoids and expressions of the key enzyme genes in the biosynthetic pathway of *Ganoderma* triterpenoids (Figure 6). On the contrary, in *GlbHLH5-*silenced *G. lucidum* by RNA interference, the triterpenoids accumulation along with the expression levels of key enzyme genes in the biosynthetic pathway were significantly and synchronously reduced (Figure 7). What’s more, the variations of the pathway genes transcription were consistent with the accumulation of *Ganoderma* triterpenoids, and the transcription of *GlbHLH5* as well, indicating *GlbHLH5* transcription factor is a positive regulator of triterpenoids biosynthesis in *G. lucidum* by activating the synergistic expression of key enzyme genes in the triterpenoid metabolism pathway, hereby a schematic of the predicted regulatory effect of *GlbHLH5* in *Ganoderma* triterpenoids biosynthesis was illustrated in Figure 8. To sum up, *GlbHLH5* is a bHLH transcription factor that positively regulates the biosynthesis of *Ganoderma* triterpenoids in response to JA, the function is similar to that of *Salvia SmMYB1* (Zhou et al., 2021), *SmbHLH53* (Peng et al., 2020), *Catharanthus BIS1* (Singh et al., 2021), *CrMYC2* (Hedhili et al., 2010), etc. It is consistent that multiple bHLH transcription factors regulate terpenoid synthesis in response to JA signals.

## Conclusion

In this study, the gene of the bHLH transcription factor *GlbHLH5* that regulates the biosynthesis of *Ganoderma* triterpenoids was cloned for the first time. The gene and protein sequence, transcription activation activity and subcellular location were systematically analyzed. Its regulation of triterpenoids synthesis was further confirmed by genetic transformation. It has been shown that *GlbHLH5* is a bHLH transcription factor which could positively regulate *Ganoderma* triterpenoids synthesis by responding to JA signal. It lays the foundation for further exploration of the molecular mechanism of metabolism regulation by *GlbHLH5* transcription factor.

## Data Availability Statement

The original contributions presented in the study are included in the article/Supplementary Material, further inquiries can be directed to the corresponding author.

## Author Contributions

JX performed a part of experiments, designed the experiments and technical route, and mainly revised the manuscript. YW performed the majority of experiments, data analysis, and drafted the manuscript. SR and XY were mainly responsible for the revision of manuscripts. YZ and KX participated in a part of experiments and helped to collect samples. XW conceived the project, revised the manuscript, and provided final approval of the manuscript. All authors read and approved the final manuscript.

## Conflict of Interest

KX was employed by Zhejiang Wuyangtang Pharmaceutical Co., Ltd. The remaining authors declare that the research was conducted in the absence of any commercial or financial relationships that could be construed as a potential conflict of interest.

## Publisher’s Note

All claims expressed in this article are solely those of the authors and do not necessarily represent those of their affiliated organizations, or those of the publisher, the editors and the reviewers. Any product that may be evaluated in this article, or claim that may be made by its manufacturer, is not guaranteed or endorsed by the publisher.

## Footnotes

1
https://www.ncbi.nlm.nih.gov/nuccore/AGAX00000000.1/
