# Supplementary material for: Identification of a Novel Metabolic Target for Bioactive Triterpenoids Biosynthesis in Ganoderma lucidum
Source: Front Microbiol. 2022 May 9;13:878110. doi: 10.3389/fmicb.2022.878110 (PMC9125208; doi:10.3389/fmicb.2022.878110)
Supplement: Supplementary file 1 [file Table_1.DOCX]

**Table S1. 1 PCR primers**

| Gene | Sequences (5’-3’) |
| --- | --- |
| GlbHLH5 | F: ATGGATCAGCACCAGCAGCA |
|  | R: ATAGCCATTTTCCGACGCAG |
| GlbHLH5-G | F: GACCTCGACTCTAGAGGATCCATGGATCAGCACCAGCAGC |
|  | R: CTTTACTCATGCTTCCACCGCCACCATAGCCATTTTCCGACGCAG |
| L-GFP | F: GGCTATGGTGGCGGTGGAAGCATGAGTAAAGGAGAAGAACTTTTCA  R: GATAAGCTTGATATCGAATTCTTATTTGTATAGTTCATCCATGCCA |
| GFP | F: CGACTCTAGAGGATCCATGAGTAAAGGAGAAGAACTTTTCA |
|  | R: GATAAGCTTGATATCGAATTCTTATTTGTATAGTTCATCCATGCCA |
| GlbHLH5-BD | F: CATATGGCCATGGAGGCCGAATTCATGGATCAGCACCAGCAGC |
|  | R: GCAGGTCGACGGATCCATAGCCATTTTCCGACGCAG |
| pBA-5 | F: TCCCCCGGGCTGCAGGAATTCATGGATCAGCACCAGCAGC |
|  | R: GGACCGGACGGGGCGGTACCATAGCCATTTTCCGACGCA |
| pSi-1 | F: CCGCTCGAGCGGATGTACCCTTCATCAGAGGACGATACC |
|  | R: CCCAAGCTTGGGATGCTGAAAGTGGCATTGCACCA |
| pSi-2 | F: GGGGTACCCCATGTACCCTTCATCAGAGGACGATACC |
|  | R: ACATGCATGCATGTATGCTGAAAGTGGCATTGCACCA |
| q-5 | F: TACGCGAGATTGAAAGATGTCCT |
|  | R: TTGGTCGATATTTGACGATTGCG |
| HMGR | F: CATCACAGCGGTTACTGCTAATG |
|  | R: GTGAGGAAAGAGGCGATCAAAAG |
| SQS | F: TTTCTCTGGGGTCATCAAGGAAG |
|  | R: GCATCTTTTCCGCAATGTCGATA |
| LS | F: CAAGATGATGAACCAGATCGTGC |
|  | R: CTCCCTGATCTGGCAATTATCCA |

Note: The thick underlines indicate restriction sites.
